# Data management system for diabetes clinical trials: a pre-post evaluation study

**DOI:** 10.1186/s12911-023-02110-w

**Published:** 2023-01-20

**Authors:** Aynaz Nourani, Haleh Ayatollahi, Masoud Solaymani-Dodaran

**Affiliations:** 1 Department of Health Information Technology, Urmia University of Medical Sciences, Urmia, Iran; 2 Health Management and Economics Research Center, Health Management Research Institute, Iran University of Medical Sciences, Tehran, Iran; 3 Department of Health Information Management, School of Health Management and Information Sciences, Iran University of Medical Sciences, Tehran, Iran; 4 Department of Epidemiology, Iran University of Medical Sciences, Tehran, Iran

**Keywords:** Data management system, Clinical trial, Diabetes, Pre- post evaluation, Usability

## Abstract

**Background:**

Data management system for diabetes clinical trials is used to support clinical data management processes. The purpose of this study was to evaluate the quality and usability of this system from the users’ perspectives.

**Methods:**

This study was conducted in 2020, and the pre-post evaluation method was used to examine the quality and usability of the designed system. Initially, a questionnaire was designed and distributed among the researchers who were involved in the diabetes clinical trials (n = 30) to investigate their expectations. Then, the researchers were asked to use the system and explain their perspectives about it by completing two questionnaires.

**Results:**

There was no statistically significant differences between the users’ perspectives about the information quality, service quality, achievements, and communication before and after using the system. However, in terms of the system quality (*P* = 0.042) and users’ autonomy (*P* = 0.026), the users’ expectations were greater than the system performance. The system usability was at a good level based on the users’ opinions.

**Conclusion:**

It seems that the designed system largely met the users’ expectations in most areas. However, the system quality and users’ autonomy need further attentions. In addition, the system should be used in multicenter trials and re-evaluated by a larger group of users.

## Introduction

The complexity of clinical trials is usually considered an influencing factor which makes managing research processes difficult [1]. In clinical trials, clinical data management is defined as planning, implementing, and monitoring policies for collecting, controlling, protecting, presenting, and enhancing the value of data and information assets in the field of clinical trials [2]. In general, the clinical data management process is a multifaceted process including designing case reports, annotating forms, creating databases, entering data, validating data, managing discrepancies and resolving data disputes, medical coding, data mining, database locking, documenting data management processes, and maintaining data security during the study [2–5]. In this process, many people such as researchers, data entry operators, data analysts, managers, and clinical trial supervisors are involved [2–6].

It should be noted that in clinical research, especially in the longitudinal studies such as clinical trials, data collection and management is a sensitive and error-prone process that can affect the final results of the study. Moreover, due to the diversity of people involved in the clinical data management and complexity of the related processes, the use of information and communication technologies, especially clinical data management systems, seems necessary [7–11].

The use of clinical data management systems is an effective solution to manage clinical data properly and improve data completeness and accuracy [1, 7, 11]. In the past, clinical data management processes were paper-based, centralized, and performed manually. In addition, when the participant was clinically examined, data were documented in the case report forms [12]. These forms were then sent to the clinical trial coordination center to be entered into the spreadsheets such as EXCEL or SPSS [13, 14]. To retrieve the data and resolve any inconsistencies in the documented data, one could write a request on a paper form and send it to the clinical centers. Finally, the clinical trial manager made the corrections and added notes on the same form to be used in the databases [12].

To overcome the challenges of paper-based processes, clinical data management systems were used as a modern and reliable solution to manage clinical trial data more effectively [7]. The most important potentials of these systems are facilitating the process of design and distribution of case report forms, facilitating data collection and documentation, reducing data entry errors, and protecting data security [15–17]. These functions can reduce the workload of the researchers and increase confidence in reporting the research results [5, 18]. However, many studies indicate that sometimes information systems, including clinical data management systems, may not have positive outcomes, and in some cases, an increased workload and user dissatisfaction have been reported [16, 19–21]. Sometimes users may resist using the new systems, mainly due to the technical issues, such as interface design that should be addressed during the system design, and the non-technical issues, such as the gap between the users’ expectations and their experiences of using new information systems [22–24]. Although many other factors such as organizational context and insufficient staff training may also influence the future use of the systems [23, 24], the literature shows that user’s expectations before and after using the technology play an important role in determining the level of user satisfaction, and there is a significant positive relationship between ‘system usage’ and ‘user satisfaction’ [22]. Therefore, evaluation of information systems, including clinical data management systems is necessary, especially from the users’ points of view [25].

There are several methods for evaluating clinical data management systems, and the choice of which depends on the purpose of the evaluation [26–28]. One of the most important evaluations for most clinical data management systems is the evaluation of system quality and usability [26], which can be conducted by obtaining user feedback before and after system usage (pre-post evaluation study). This type of evaluation can be divided into several categories based on the goals of the researchers: (a) evaluation of a system before and after using it to check the compliance of the system performance with the users’ expectations, (b) evaluation of two systems to compare their functions and select a more appropriate one, and (c) evaluation of several systems to compare their performance and rank them [29]. It is worth noting that conducting an evaluation study before and after using a system can be an effective method to obtain user feedback and measure their expectations [30]. This type of evaluation leads to more contacts with the real users of the system and is an advantage for the precise understanding of the system performance [31]. In fact, a good technical design and willingness of users to use the new technology are critical factors for the successful implementation of clinical information systems [22].

**Fig. 1:**
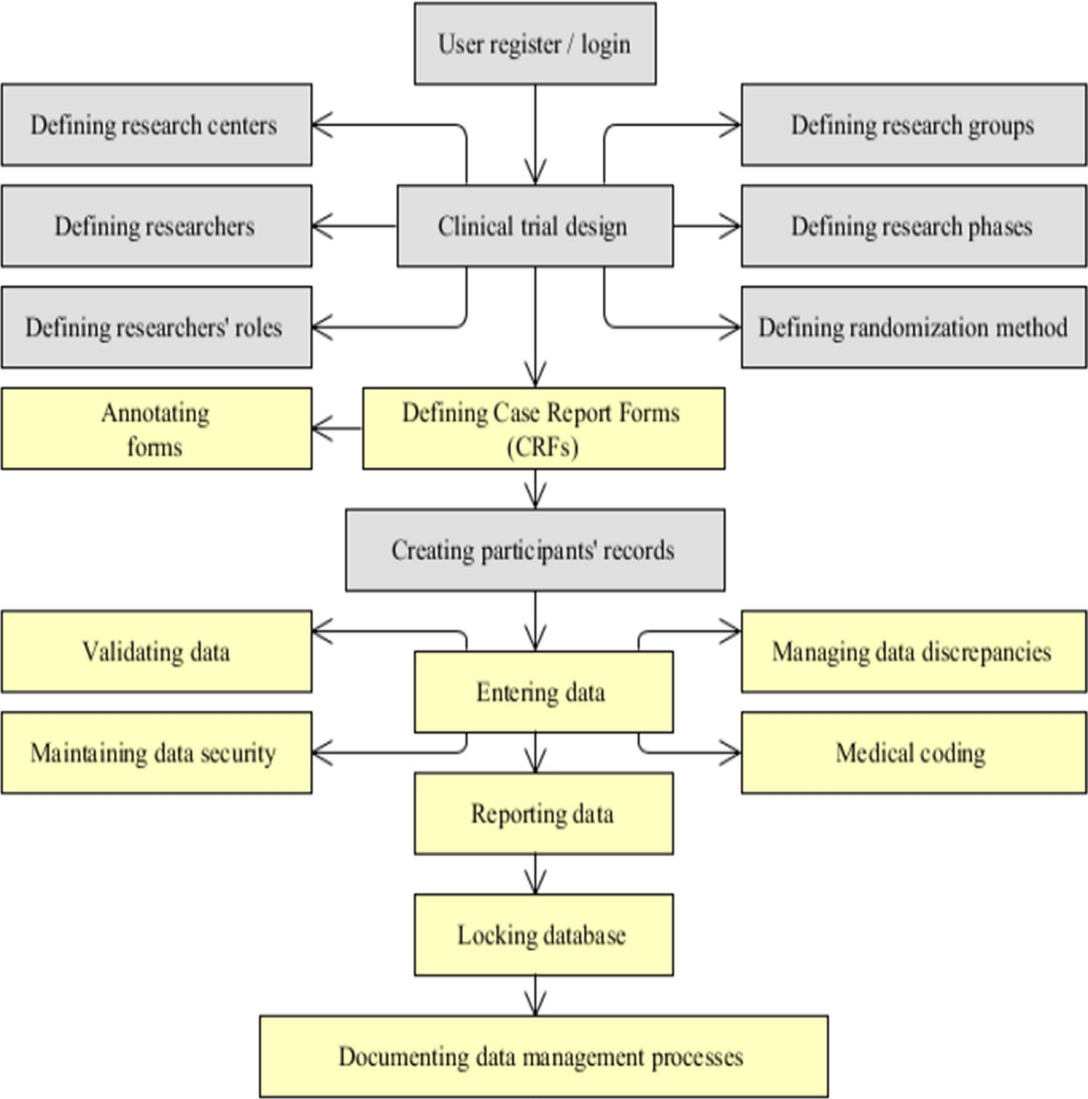
Main functions of the diabetes clinical trials data management system; Gray boxes: Clinical trial activities; Yellow boxes: Data management activities

Previously, a clinical data management system was designed for diabetes clinical trials in Iran [32]. The purpose of this system was to provide a regular and electronic process for data management in diabetes clinical trials. As Fig. 1 shows, the main functions of the system were designing clinical trials, defining users’ roles, creating case report forms, auditing, documenting clinical data management process, reviewing participants’ records, generating reports and statistics, locking databases, and backing to the profile. In addition, a clinical trial manger was able to define a clinical trial setting, design case report forms, annotate forms, create databases, validate data, manage data discrepancies, resolve data disputes, and maintain data security [32]. The purpose of this study was to evaluate the quality and usability of this system from the users’ perspectives.

## Methods

This quantitative study was conducted in 2020. The participants were the researchers who were involved in diabetes clinical trials and worked in two endocrinology and metabolism research centers affiliated to the medical universities (n = 30). The purposive sampling method was used to select the participants who have at least two years of work experience in conducting diabetes clinical trials as the main investigator, supervisor, and research partner.

## Research instruments

In this study, two questionnaires were used to collect data [30, 33]. The pre-post evaluation of the clinical data management system for diabetes clinical trials was performed using the questionnaire developed by Karimi et al [30]. This questionnaire was designed to determine the users’ expectations before using the system and the level of their satisfaction and expectation fulfillment after using the system. It was a seven-point Likert scale questionnaire composed of six sections and included information quality (four questions), system quality (five questions), service quality (two questions), achievements (four questions), communications (four questions), and user autonomy (six questions). The wording of the questions was presented in a way that could be used before and after using the system. The reliability of the questionnaire was assessed by calculating the internal correlation coefficient (Cronbach’s alpha) ($\alpha =0.98$).

To evaluate the usability of the system, the questionnaire for user interaction satisfaction (QUIS), version 7.0 was used [33]. It was a 10-point Likert scale questionnaire and had 32 questions that were organized in six sections, overall reaction to the system (six questions), screen design and layout (four questions), terminology and system information (six questions), learning (six questions), system capabilities (five questions), and usability and user interface (five questions). The reliability of the QUIS questionnaire was calculated using Cronbach’s alpha ($\alpha =0.91$).

### Data analysis

Data were analyzed using descriptive statistics (mean value and standard deviation). Kolmogorov–Smirnov test was used to assess the normality of data distribution and the mean values were compared using the non-parametric Wilcoxon test. To determine the usability of the system, the score of the Likert scale (0–9), was divided into three categories. The mean value between zero and 3 indicated a poor level of the system usability, the mean value between 3.1 and 6 indicated an intermediate level of the system usability, and the mean value between 6.1 and 9 showed a good level of the system usability from the users’ perspectives.

## Results

### Participants’ characteristics

As Table 1 shows a total of 30 researchers experienced in conducting diabetes clinical trials participated in this study and most of them were female (n = 22, 73.3%). The highest frequency (n = 12, 40.0%) belonged to the age range of 36–45 years old, and about half of the participants (n = 14, 46.7%) were specialists in endocrine and metabolic diseases.

**Table 1:** Participants’ characteristics

Variables		Frequency (percentage)
Sex	Male	8 (26.7%)
	Female	22 (73.3%)
Age	26–35	7 (23.3%)
	36–45	12 (40.0%)
	46–55	9 (30.0%)
	56–65	2 (6.7%)
Educational level	M.D.	16 (53.3%)
	Ph.D.	13 (43.3%)
	M.Sc.	1 (3.3%)
Field of study	Endocrine and metabolic diseases	14 (46.7%)
	Nutrition	7 (23.3%)
	Epidemiology	3 (10.0%)
	Obstetrics and infertility	2 (6.7%)
	Pregnancy health	4 (13.3%)
Work experience (years)	2–8	11 (36.7%)
	9–15	13 (43.4%)
	16–22	4 (13.3%)
	23–29	1 (3.3%)
	30–37	1 (3.3%)

## Pre-post evaluation study

Initially, the pre-evaluation questionnaire was administered to 30 researchers. These people had not seen or used the system before completing the questionnaire and had no knowledge of its performance and capabilities. Therefore, they merely expressed their expectations about the system and the results showed that the mean values for the users’ expectations were as follows: information quality (7.0 ± 0.0), system quality (7.0 ± 0.0), service quality (6.6 ± 0.33), achievements (6.9 ± 0.15), communications (6.8 ± 0.40), and users’ autonomy (6.3 ± 1.05).

Then, the researchers were provided by guidelines on how to access to the system, and were asked to use the system for one to two weeks. After this time, they were asked to complete the post-evaluation questionnaire. The findings showed that the mean values for different parts of the questionnaire were as follows: information quality (5.5 ± 1.18), system quality (5.8 ± 1.12), service quality (5.1 ± 1.17), achievements (5.4 ± 1.25), communications (5.4 ± 1.2), and users’ autonomy (5.7 ± 1.24). Tables 2 and 3 show the participants’ perspectives about users’ autonomy before and after using the system.

**Table 2:** Participants’ responses regarding their expectations of users’ autonomy before using the system

Users’ autonomy	Strongly disagree Fr (%)	Disagree Fr (%)	Slightly disagree Fr (%)	Neither Agree nor disagree Fr (%)	Slightly agree Fr (%)	Agree Fr (%)	Strongly Agree Fr (%)	Mean	SD
I desire to have control over various dimensions of clinical trial data management.	15 (50.0%)	8 (26.6%)	2 (6.7%)	5 (16.7%)	0	0	0	6.1	1.12
I desire to have control over managing research centers in a clinical trial.	19 (63.3%)	6 (20.0%)	5 (16.7%)	0	0	0	0	6.6	0.89
I desire to have control over managing all people involved in a clinical trial.	17 (56.7%)	6 (20.0%)	4 (13.3%)	2 (6.7%)	1 (3.3%)	0	0	6.2	1.13
I desire to have control over the case report forms of the participants.	16 (53.3%)	10 (33.4%)	2 (6.7%)	1 (3.3%)	1 (3.3%)	0	0	6.3	0.99
I desire to have control over the security of clinical trial data.	13 (43.4%)	10 (33.4%)	4 (13.3%)	1 (3.3%)	1 (3.3%)	1 (3.3%)	0	6.0	1.23
I desire to have control over the quality of clinical trial data.	19 (63.3%)	6 (20.0%)	5 (16.7%)	0	0	0	0	6.6	0.89
Total mean and SD								6.3	1.05

**Table 3:** Participants’ opinions about users’ autonomy after using the system

Users’ autonomy	Much more than expected Fr (%)	More than expected Fr (%)	Slightly more than expected Fr (%)	As expected Fr (%)	Slightly less than expected Fr (%)	Less than expected Fr (%)	Much less than expected Fr (%)	Mean		SD	
By using the system, how well are your needs met in terms of controlling various dimensions of clinical trial data management?	13 (43.4%)	7 (23.3%)	5 (16.7%)	5 (16.7%)	0	0	0	5.9		1.14	
By using the system, how well are your needs met in terms of managing research centers in a clinical trial?	8 (26.6%)	9 (30.0%)	7 (23.3%)	4 (13.3%)	2 (6.7%)	0	0	5.6		1.22	
By using the system, how well are your needs met in terms of managing all people involved in a clinical trial?	9 (30.0%)	8 (26.6%)	5 (16.7%)	6 (20.0%)	2 (6.7%)	0	0	5.5		1.31	
By using the system, how well are your needs met in terms of controlling case report forms of the participants?	9 (30.0%)	10 (33.4%)	3 (9.9%)	6 (20.0%)	2 (6.7%)	0	0	5.6		1.30	
By using the system, how well are your needs met in terms of controlling security of clinical trial data?	8 (26.6%)	7 (23.3%)	6 (20.0%)	7 (23.3%)	2 (6.7%)	0	0	5.4		1.36	
By using the system, how well are your needs met in terms of controlling quality of clinical trial data?	12 (40.0%)	8 (26.6%)	6 (20.0%)	4 (13.3%)	0	0	0	5.9		1.08	
Total mean and SD								5.7		1.24	

The result of the Kolmogorov–Smirnov statistical test revealed that the distribution of data before using the system was not normal (*P* = 0.00); however, it was normal after using the system (*P* = 0.20). Therefore, the non-parametric Wilcoxon test was used to examine any statistically significant differences between the users’ expectations before and after using the system (Table 4).

**Table 4:** Users’ expectations before and after using the system

Questionnaire’s sections		Users’ expectations before using the system		Users’ expectations after using the system	
		Mean	SD	Mean	SD
*Information quality*					
1	Reliability	7.0	0.0	5.5	1.17
2	Up-to-date data	7.0	0.0	5.2	1.38
3	Relevancy	7.0	0.0	5.7	1.06
4	User-friendliness	7.0	0.0	5.6	1.10
Total mean and SD		7.0	0.0	5.5	1.18
		(*P* value = 0.068)			
*System quality*					
5	System reliability	7.0	0.0	5.6	1.22
6	System flexibility	7.0	0.0	5.6	1.14
7	High speed	7.0	0.0	5.9	0.90
8	Ease of use	7.0	0.0	5.5	1.28
9	Ease of access	7.0	0.0	6.3	1.05
Total mean and SD		7.0	0.0	5.8	1.12
		(*P* value = 0.042)			
*Service quality*					
10	Immediate IT support services	6.8	0.35	5.1	1.17
11	Comprehensive IT support services	6.9	0.31	5.1	1.17
Total mean and SD		6.6	0.33	5.1	1.17
		(*P* value = 0.180)			
*Achievements*					
12	Maximum efficiency in clinical trial data management	7	0.0	5.2	1.24
13	Few errors in managing clinical trial data	7	0.0	5.3	1.34
14	High quality clinical trial data management	6.9	0.26	5.7	1.18
15	Saving time	6.8	0.35	5.5	1.22
Total mean and SD		6.9	0.15	5.4	1.25
		(*P* value = 0.068)			
*Communications*					
16	Unambiguous communication with colleagues to get the necessary information for performing tasks	6.8	0.41	5.1	1.31
17	Efficient interaction with colleagues to get the necessary information for performing tasks	6.8	0.41	5.7	1.05
18	Hassle-free communication with colleagues to get the necessary information for performing tasks	6.8	0.41	5.5	1.14
19	Effective interaction with colleagues to get the necessary information for performing tasks.	6.8	0.38	5.2	1.30
Total mean and SD		6.8	0.40	5.4	1.20
		(*P* value = 0.068)			
*Autonomy*					
20	Controlling different dimensions of clinical trial data management	6.1	1.12	5.9	1.14
21	Managing all research centers	6.6	0.89	5.6	1.22
22	Managing all people involved in a clinical trail	6.2	1.13	5.5	1.31
23	Managing participants’ case report forms	6.3	0.99	5.6	1.30
24	Managing clinical trial data security	6.0	1.26	5.4	1.36
25	Managing clinical trial data quality	6.6	0.89	5.9	1.08
Total mean and SD		6.3	1.05	5.7	1.24
		(*P* value = 0.026)			

**Fig. 2:**
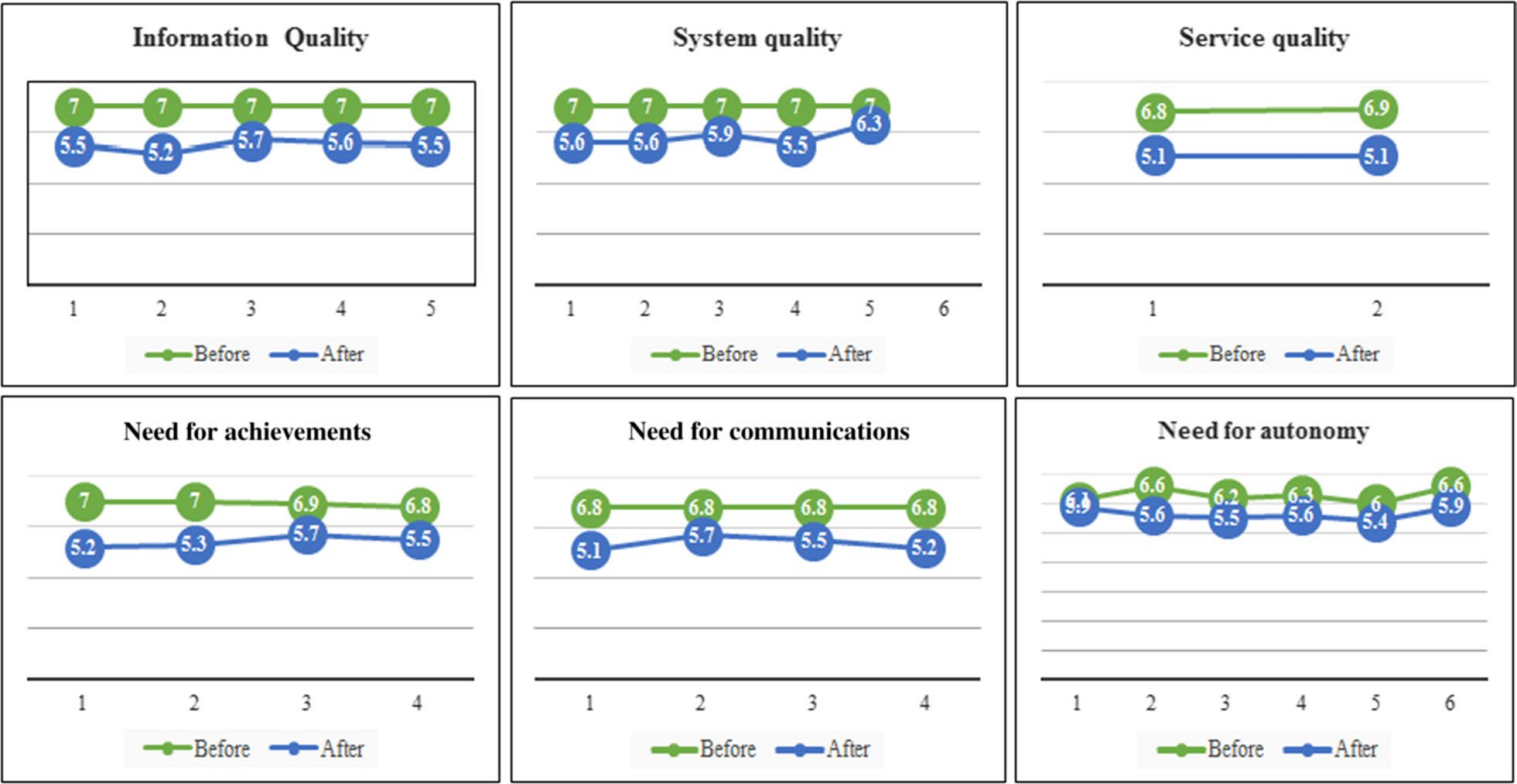
Comparison between the mean values of the users’ expectations before and after using the system

As shown in Table 4, there was no statistically significant differences between the mean values of users’ expectations before and after using the system in four areas: information quality (*P* value = 0.068), service quality (*P* value = 0.042), achievements (*P* value = 0.068), and communications (*P* value = 0.068). Therefore, it can be said that the expectations of the users were not much different from what they experienced after using the system and their expectations were met. However, there was statistically significant differences between the users’ expectations of the system quality (*P* = 0.042) and users’ autonomy (*P* = 0.026) before and after using the system. It seems that in these two areas, users’ expectations were greater than the system capabilities. A comparison between the mean values of the users’ expectations before and after using the system has been shown in Fig. 2.

## Usability evaluation

One of the most important issues in evaluating information systems is usability evaluation, which can be done by investigating users’ or experts’ perspectives [34]. In this study, the usability of the clinical data management system for diabetes clinical trials was evaluated from the users’ perspectives using the QUIS questionnaire [33]. The results of this evaluation study are presented in Table 5.

**Table 5:** Users’ perspectives about the system usability

Evaluation areas	Mean ± SD
Overall reaction to the system	7.8 ± 1.1
Screen design and layout	7.9 ± 1.1
Terminology and system information	7.6 ± 1.2
Learning	7.7 ± 1.6
System capabilities	7.6 ± 1.4
Usability and user interface	7.7 ± 1.4

According to the results, the least mean values belonged to the terminology and system information (7.6 ± 1.2) and system capabilities (7.6 ± 1.4), and the highest mean value was related to screen design and layout (7.9 ± 1.1). As all of the mean values were between 6.1 and 9, it was concluded that the most users evaluated the usability of the system at a good level.

## Discussion

Data management of clinical trials is a complex process that can be facilitated by using information and communication technologies (ICT) [35]. Clinical data management systems are technologies that can play an effective role in clinical data management, especially in multicenter clinical trials [32]. These systems support various aspects of data management, reduce financial and manpower costs, and facilitate data collection and management by eliminating manual processes and reducing workload [32, 35, 36]. However, many systems have not been properly evaluated in terms of quality, usability, and impact [21, 37–42]. Therefore, conducting evaluation studies is of paramount importance [16].

In the present study, quality and usability of a previously developed clinical data management system for diabetes clinical trials were evaluated [32]. The results revealed that there was no statistically significant differences between the users’ expectations of “information quality”, “service quality”, “achievements” and “communications” before and after using the system and their expectations were met. However, in terms of the “system quality” and “users’ autonomy”, there were statistically significant differences between the users’ expectations before and after using the system. It seems that in these two areas, the quality of the system was lower than the users’ expectations. Although users often have high and unrealistic expectations before using new systems [43, 44], the possible weaknesses of the designed systems should not be underestimated. After upgrading systems, other evaluation methods can be used to see whether users’ expectations have been met or not.

Similar to the current research, the pre-post evaluation method has also been used by other researchers who developed clinical data management systems [21, 42, 45]. For example, Wilson et al. conducted a pre-post evaluation study on the effectiveness and usability of Vasculitis Integrated Clinical Assessment Database (VICAD) and compared the results before and after using the system. The results demonstrated that VICAD was an effective system for data management. The usability evaluation of this system also showed that VICAD improved clinical assessments from 77 to 98% [21]. While in Wilson et al.’s study, the benefits of using the system and its effectiveness on the quality of work were investigated, in the present study, users’ opinions about the quality of the system were inspected.

Tran et al. developed “OnWARD” as an ontology-based web framework for multicenter clinical studies and collected users’ opinions about the flexibility, effectiveness, and ease of use before and after using the system. The preliminary results of the evaluation suggested that the flexibility, effectiveness, and ease of use were greater than the users’ expectations and the users were able to perform all data entry tasks with minimal training. In fact, the system was able to meet the staff and researchers’ requirements in multicenter clinical trial data management [42].

In another study, Müller et al. assessed the cost of clinical data management before and after using the system. In this study, the costs of manual data management before implementing the system (i.e., printing case report forms, distributing them among the researchers in different geographical areas, collecting forms, checking data quality by human resources, returning forms to the research centers in case of errors in the documented data, retrieving data from paper-based forms and re-entering them into the statistical software) were compared with the costs of data management after using the clinical data management system. The results showed that the use of the computer-based system significantly reduced the cost of clinical data management [45].

In the second phase of the present study, the usability of the system was evaluated based on the users’ perspectives. The mean values revealed that the users evaluated the usability of the system at a “good” level. Although usability evaluation is important and can directly influence user satisfaction, it has not been reported in other similar studies or they used other methods or questionnaires to investigate users’ opinions [15–17, 21, 28, 37–42, 45–52]. Overall, we can conclude that the system designed in the current study can be improved based on the participants’ perspectives, and can be implemented in a real environment to examine how it can work to meet the users’ requirements in a clinical trial.

## Research limitations

As the current clinical data management system for diabetes clinical trials has not yet been approved by the national office of health technology in the Ministry of Health, in the current study, the real data were not entered into the system to maintain the confidentiality issues. Moreover, a limited number of the researchers participated in this study. This might be due to the limited number of the researchers who had at least two years of work experience in conducting diabetes clinical trials as the main investigator, supervisor, and research partner, or the lack of interest about the subject of the research. As a result, we were not able to present the findings based on different specialties. Therefore, evaluating the system in a larger population is recommended.

## Conclusion

The aim of this study was to evaluate the quality and usability of a clinical data management system for diabetes clinical trials from the users’ perspectives. The findings of this study showed that the designed system was able to meet the users’ expectations in most areas. In addition, users evaluated the usability of the system at a good level. Therefore, it seems that the system has been tailored to the users’ requirements and can help them to conduct future clinical trials in a more systematic way, which in turn helps to improve efficiency and effectiveness of clinical data management. To be able to implement and use the system at the national level, conducting future studies in a larger sample size by using other evaluation methods is suggested.

## Data Availability

All data generated or analyzed during this study are included in this article.

## Abbreviations

ICT: Information and Communication Technology
VICAD: Vasculitis Integrated Clinical Assessment Database
QUIS: Questionnaire for User Interaction Satisfaction
